# Bridging the knowledge-practice gap in tuberculosis contact management in a high-burden setting: a mixed-methods protocol for a multicenter health system strengthening study

**DOI:** 10.1186/s13012-019-0870-x

**Published:** 2019-03-19

**Authors:** Trisasi Lestari, Steve Graham, Christel van den Boogard, Rina Triasih, Jeanne Rini Poespoprodjo, Reynold Rizal Ubra, Enny Kenangalem, Yodi Mahendradhata, Nicholas M. Anstey, Ross S. Bailie, Anna P. Ralph

**Affiliations:** 1 Global and Tropical Health, Menzies School of Health Research, Charles Darwin University, Darwin, Northern Territory Australia; 2 Centre for International Child Health, Department of Paediatrics and Murdoch Children’s Research Institute, University of Melbourne, Melbourne, Australia; 3 Burnet Institute, Melbourne, Australia; 4 Center for Tropical Medicine, Faculty of Medicine, Public Health, and Nursing, Universitas Gadjah Mada, Yogyakarta, Indonesia; 5 Papuan Health and Community Development Foundation, Papua, Indonesia; 6 Mimika District Health Authority, Papua, Indonesia; 7 Division of Medicine, Royal Darwin Hospital, Darwin, Northern Territory Australia; 8 University Centre for Rural Health, School of Medicine, University of Sydney, Lismore, Australia

**Keywords:** Tuberculosis contact investigation, Preventive treatment, Consolidated Framework for Implementation Research, Behaviour Change Wheel, RE-AIM model

## Abstract

**Background:**

People in close contact with tuberculosis should have screening and appropriate management, as an opportunity for active case detection and prevention. However, implementation of tuberculosis contact screening and management is limited in high-burden settings. Behaviour change is needed across three levels of the healthcare system—policymakers, healthcare providers, and patients. To bridge the wide policy-practice gap, this study draws on the Consolidated Framework for Implementation Research, the Behaviour Change Wheel, and the RE-AIM model (Reach, Effectiveness, Adoption, Implementation, Maintenance) to respectively understand barriers, implement change, and evaluate process and outcome.

**Methods:**

This methods paper describes a mixed-methods intervention study in Eastern Indonesia. Quantitative data will be collected during baseline, intervention, and sustainability periods and analyzed using time series analysis. The primary outcome is the number of individuals completing tuberculosis preventive therapy by the end of the two-year intervention phase. Of an estimated 10,000 contacts during this period, we anticipate that a minimum of 416 will be found to have active TB or will complete preventive therapy. Qualitative data (semi-structured interviews, focus group discussions, and observations) will be collected from consenting healthcare providers, patients, and contacts. Activities to promote policy implementation include healthcare provider training, quarterly continuous quality improvement workshops, a social media discussion forum, and promotional materials. The Consolidated Framework for Implementation Research will be used to identify reasons for limited policy implementation at baseline. The Behaviour Change Wheel will be used to ensure that a suitable range of activities are implemented to facilitate change. The RE-AIM model will be used as the evaluation framework.

**Discussion:**

Use of theoretical frameworks in combination can ensure a comprehensive understanding of, and robust response to, health policy underimplementation. The selected frameworks are highly applicable to this pragmatic intervention study, in a setting where End TB Strategy targets will not be met without substantial behavior change within health systems. Continuous quality improvement cycles will provide a way to engage staff and stakeholders in understanding local data to motivate behavior change. If successful, up to 500 people could be prevented from developing complications of tuberculosis through early case-finding or receiving preventive therapy over a two-year period.

**Study registration:**

Australian New Zealand Clinical Trials Register 375803.

## Background

The World Health Organization (WHO) has set ambitious targets for global tuberculosis (TB) control through the End TB Strategy [1]. TB is the leading infectious cause of death globally [2]. The strategy emphasizes TB prevention and research to optimize implementation [3]. A key TB prevention activity comprises contact investigation and management for people living in the same household as someone with infectious TB. Contact investigation provides opportunities to detect co-prevalent TB—that is, other people with active TB (“TB disease”) who might have been the source or have acquired infection from the index case. It also allows people with latent infection (“TB infection”) to be detected and commenced on treatment to prevent incident disease [4].

WHO guidelines strongly recommend that after excluding active TB, high-risk contacts of confirmed pulmonary TB cases be commenced on preventive therapy. Those at highest risk are young children (aged < 5 years) and people of any age with immunosuppression [5]. A number of effective preventive treatment regimens are recommended [5], the most commonly recommended being isoniazid as a daily oral medication for 6 months, known as isoniazid preventive therapy (IPT).

IPT effectiveness is described in a systematic review of 11 trials involving > 70,000 patients. This concluded that one person can be prevented from getting TB when 35 contacts take isoniazid for at least 6 months [6]. The potential benefits are greatest in young children because of their high risk of developing TB disease after exposure [7–9]. Contact investigation and management, incorporating TB preventive therapy plus early detection of active disease, is a highly cost-effective strategy, and cost-effectiveness can be further increased by using a symptom-based screening approach [10]. Symptom-based screening avoids the need for diagnostic tests for TB infection (the tuberculin skin test or an interferon gamma release assay) and investigations to exclude active disease (e.g., chest radiograph), which are often difficult to access in low-resource settings. Symptom-based screening has been shown in Indonesian children to be a safe and effective way of determining which child contacts require further evaluation for TB disease, and which can be commenced on TB preventive therapy in the community without the need for further evaluation [11, 12].

Despite the compelling case and universal recommendations for TB contact management, a major policy-practice gap exists in low-resource, high TB-burden settings. A variety of well-identified barriers at health system and patient levels contribute to the poor implementation of contact management globally [7, 13–15]. Commonly identified barriers include the distance to clinics or cost of travel, time away from work, or lack of understanding of the value of contact screening among healthcare providers or asymptomatic contacts [14–16]. New strategies have been identified that can be applied to close this gap [9]. The main requirement to successfully embed this policy is behavior change at the levels of health systems, healthcare providers, and contacts and their families.

### Specific challenges in Indonesia

Indonesia is among the top three highest TB-burden countries internationally [2]. The results of a national TB prevalence survey revealed that TB prevalence among Indonesian adults was almost double previous estimates (759 vs 391 per 100,000 population) [17]. A previous evaluation of the implementation of child contact management in Bandung, Java, reported wide implementation gaps in the cascade of care with only 8% (34/437) of child contacts presenting for screening within 3 months of adult index case diagnosis [18]. This low uptake is similar to findings from elsewhere when contact management is “passive” and facility-based [19–21]. Now, an ambitious international target has been set for ≥ 90% of young TB-exposed children to receive preventive treatment by 2035 [22]. In response to this call, the Indonesian National TB Program has embraced contact management and investigation as an important component of the Indonesian TB program [1, 8, 23].

### Implementation science approaches in TB research

Too often, deployment of a new policy relies on the distribution of a clinical practice guideline without provision of adequate staff training, with resulting inadequate uptake. A systematic method incorporating knowledge of the barriers which need to be addressed, the behaviors which need to be changed, and strategies likely to be effective is needed to improve intervention design [24]. Validated theoretical frameworks and implementation strategies can be used in projects designed to bridge the policy-practice gap in TB contact management [25].

There is a strong culture of “operational research” in TB, sometimes used interchangeably with the term “implementation research” [26], to improve program performance [27]. But surprisingly, there is limited reported use of theory-derived frameworks in TB research. The Behaviour Change Wheel [24] has been applied to TB contact investigation in Uganda and found to be an effective strategy for identifying barriers to and facilitators of this policy [13]. The theory of planned behavior and the PRECEED model (“Predisposing, Reinforcing and Enabling Constructs in Educational Diagnosis and Evaluation”) have been successfully applied to improve the quality of TB care, also in Uganda [28]. However, few other studies report the application of theoretical frameworks in TB operational research. A systematic review identified that theoretical frameworks to underpin behavior-change interventions have been underutilized in general in the past [29]. There are increasing calls to research frameworks in these contexts to ensure that interventions are comprehensive, well-structured, and replicable [30].

In our study to implement contact management in Mimika district, Eastern Indonesia, we use the Consolidated Framework for Implementation Research (CFIR) [31] to identify current reasons for the lack of policy implementation and the COM-B model (“Capability, Opportunity, and Motivation Behaviour system”) of the Behaviour Change Wheel [24] as a means of categorizing activities required for successful policy implementation. We use the RE-AIM model (Reach, Effectiveness, Adoption, Implementation, Maintenance) [32, 33] as the evaluation framework.

The overarching objective of this health system strengthening research is to reduce TB rates in an under-resourced, high-burden setting, especially among infants and young children, by effectively implementing TB contact management. To facilitate policy implementation, change is required across three levels of the healthcare system—policymakers, healthcare providers, and patients and their families. The aim of this methods paper is to describe the protocol for this study, including theoretical underpinnings and methodological approaches. We describe context-specific barriers to and facilitators of TB contact management at the study site using the CFIR and map these to the Behaviour Change Wheel.

## Methods

This is a mixed-methods, before-and-after intervention study, using interrupted time series (ITS) analysis. Activities fulfill the definition of a complex intervention (Table 1) [34]. A suite of interventions will be implemented in a staggered way over 24 months, independent of other changes in the TB program. The study is led by a collaborative investigator group in Indonesia and Australia and registered with the Australian New Zealand Clinical Trials Registry (trial ID: ACTRN12618001503213) [35]. At the field research site, two study nurses are employed as research assistants and data managers. They are supervised by a local medical doctor currently enrolled as a doctoral research scholar (TL) who lives on site as the main study implementer. The strength of relationships between the research team with stakeholders and healthcare providers contributes importantly to feasibility.

**Table 1:** Alignment of study activities with Medical Research Council UK measures of intervention complexity

Characteristic of activities	Extent to which the study activities are complex
Number of interacting components within the experimental and control interventions	Activities include: **•** Education and training **•** Continuous quality improvement workshops **•** Provision of medication There is a retrospective control period and a prospective experimental period.
Number and difficulty of behaviors required by those delivering or receiving the intervention	Health care providers need to significantly change practice to start doing contact investigation, which was not previously undertaken: **•** Details of contacts need to be documented on a new TB form. **•** Contacts need to be seen by healthcare providers, assessed, investigated appropriately, commenced on medication as appropriate, and followed up for at least 6 months. **•** Patients need to adhere to medication and medical follow-up appointments.
Number of groups or organizational levels targeted by the intervention	Five healthcare facilities (phase 1 implementation) Seven healthcare facilities (phase 2 implementation) District TB Program District Health Authority
Number and variability of outcomes	There are six main outcomes: Primary: **•** Number of individuals completing isoniazid preventive therapy (IPT). Secondary (numbers and proportions of): **•** Index cases for whom contact investigation and management is undertaken **◦** All **◦** Smear-positive **◦** Smear-negative **•** Contacts reached for symptom screening **•** Contacts found to have active TB **•** Contacts eligible for IPT **•** Contacts commencing IPT.
Degree of flexibility or tailoring of the intervention permitted	A degree of tailoring is permitted since healthcare facilities differ (hospitals, primary care sites, rural, urban, etc.)

Factors listed in the left hand column are derived from Craig et al [34]

### Setting

Mimika district, population 201,677, spans from the coastal to highland areas in southern Papua Province, Indonesia [36]. Mimika is characterized by ethnic contrasts, challenges of multiple languages, geographical isolation of remote communities, poverty, and poor health outcomes [36, 37]. Proceeds from a large gold and copper mine fund some private clinics and hospitals. The estimated incidence of notified active TB cases in Mimika district in 2017 was 743/100,000, compared with the national incidence of 391/100,000 [17]. HIV-TB coinfections and late presentation with advanced disease are both common [38, 39]. The estimated proportion of TB cases that are multidrug resistant (MDR) has risen from 1.3% in a study of pulmonary TB patients with initial presentations in 2008–2010 [40] to 2.4% among new cases and 13% among retreatment cases in 2018 [2].

### Model for clinic participation in study implementation

**Fig. 1:**
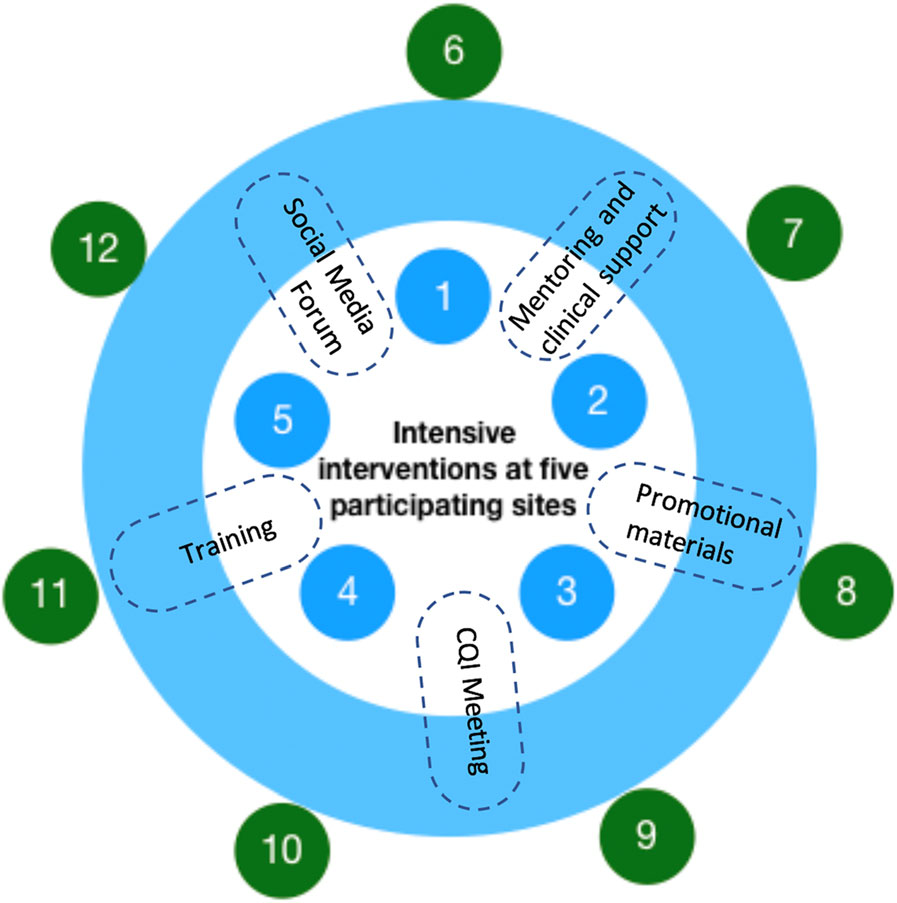
Conceptual diagram of study implementation. Intensive intervention is delivered at the five initial sites by the research staff. Second-tier intervention is carried out at the seven follow-up sites by staff from the primary sites

**Fig. 2:**
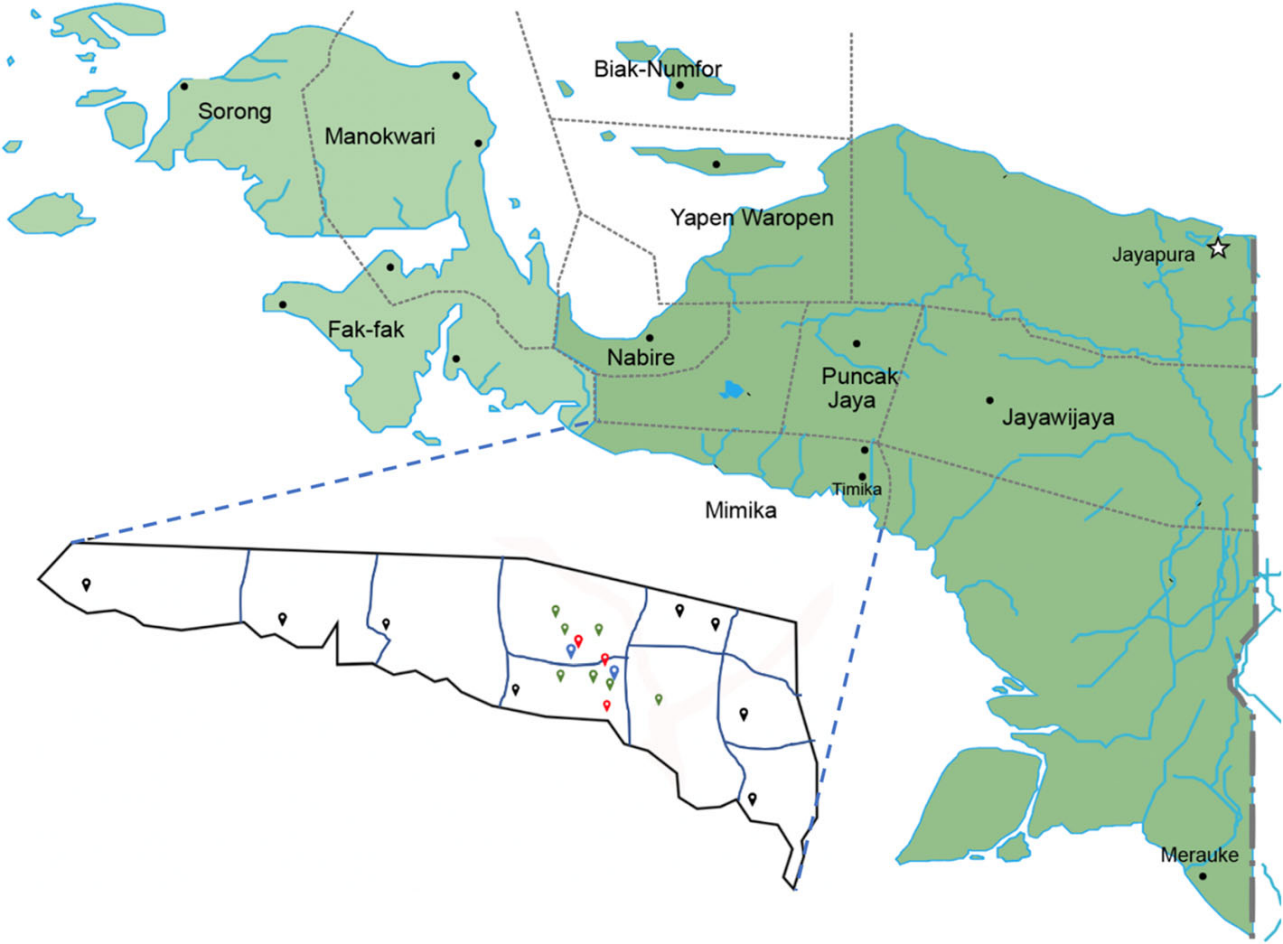
Map of Papua showing Mimika district in Papua province and participating study sites. Blue points are participating hospitals, red points are participating health centres, green points are second-phase participating health centers, and black points are non-participating centres

The study will use a two-tiered approach for policy rollout, comprising initial clinics receiving intensive intervention and follow-up clinics receiving less intensive intervention (Fig. 1). The intention is to generate a “ripple effect” from the initial sites out to a wider catchment. Of 23 primary health centers (PHCs) and 5 hospitals in Mimika district, 10 PHCs and four hospitals are authorized to provide TB diagnosis and management, free of charge. Five sites were initially invited to participate based on their TB burden and location. We purposively selected a mix of urban and rural, private and public, and primary and tertiary sites and included those with largest caseloads. All agreed to participate. Characteristics and locations are shown in Table 2 and Fig. 2. The remaining seven PHCs providing TB care in Mimika will be invited to receive a less intensive follow-up intervention.

**Table 2:** Characteristics of study sites and 2017 TB case load at the five initial participating sites

	TB case burden (2017)	Governance	Location	Number of TB nurses	Number of doctors	Number of beds
Hospital 1	228	Private	Rural	3	2	144
Hospital 2	577	Public	Urban	3	2	159
Primary healthcare center 1	47	Public	Rural	3	1	0
Primary healthcare center 2	192	Public	Urban	5	2	20
Primary healthcare center 3	43	Public	Urban	4	1	2

### Quantitative data collection and methodological approach

#### Data sources

Participating PHCs and hospitals maintain standard national data forms (Table 3), which will be used as the quantitative data for this study to complete the “cascade of care,” illustrating numbers of cases eligible for contact tracing, and losses occurring in each subsequent step. Sources and methods of data collection before and after interventions will be the same, except for additional use of new standard forms for child TB screening (TB15) and TB contact investigation (TB16) that were not available at the study sites before the intervention started. The research nurses will assist in the completion of the TB15 form initially, aiming for this task to be taken over routinely by healthcare providers as they become accustomed to it. Data of all TB cases from five initial participating centers will be collected every 2 weeks and checked for completeness. Cross-checking against original sources will be undertaken where data are incomplete, and TB staff will then be asked to complete missing data. Additional data validation will be undertaken from each participating site every 3 months to determine data reliability. Data from the seven other health facilities which receive less intensive intervention will be collected monthly.

**Table 3:** Quantitative data collection: data sources and estimated sample size during the intervention phase

Sources of quantitative data	
Form type	Name of form
Individual TB treatment card detailing diagnosis date, adherence and number of contacts	TB01
Register of all TB patients at a health facility	TB03
Laboratory register for smear sputum TB examination	TB04
Register of all people suspected of having TB	TB05
Form for child TB screening	TB15
Register of TB contacts at a health facility	TB16
Sample size	
Step of management cascade	Estimated sample size at the 5 primary sites in 24 months
Estimated number of index cases	2000
Estimated number of contacts	10,000
Estimated number of contacts reached during the study: 40%	4000
**•** Estimated number of contacts found to have co-prevalent active TB: 5%	200
**•** Estimated number likely to require TB preventive therapy (15%)	600
**◦** Estimated number commencing on IPT (60%)	360
**◦** Estimated number completing IPT (60%)	216
Estimated number of TB cases prevented through IPT	6

Individual participant consent will not be sought for quantitative data, since this is routinely collected by health services. Research staff will enter these data into an electronic database. The cleaned primary data will be double entered into Stata version 13.0. The two datasets will then be compared, differences identified and corrections made.

#### Quantitative study design

**Fig. 3:**
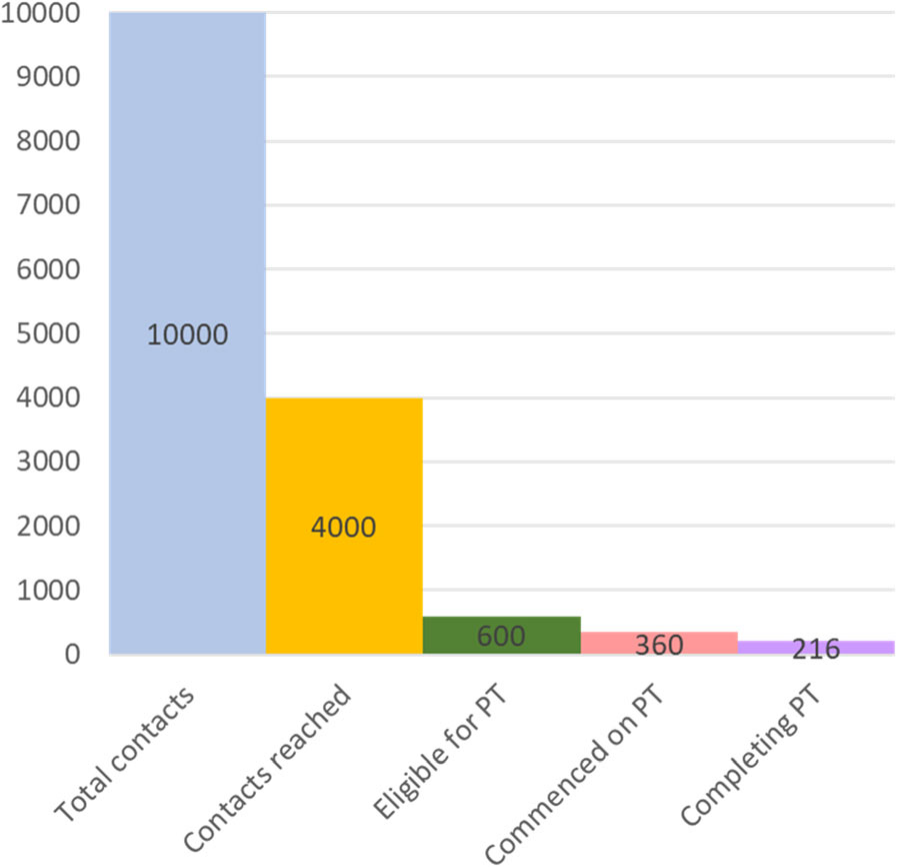
Cascade of care showing estimated numbers of contacts at each step of the cascade associated with the expected 2000 TB index cases during 24 months. *PT* preventive therapy

The primary outcome measure is the number of individuals completing IPT abstracted from individual patient records. Secondary outcomes are the successive components of the cascade of care, namely numbers and proportions of index cases for whom contact investigation and management is undertaken (sub-grouped by smear-positive and smear-negative index cases), contacts reached for symptom screening, contacts evaluated for TB disease, contacts diagnosed with TB disease, contacts treated for TB disease, contacts eligible for IPT, contacts commencing IPT, and contacts completing IPT. During the available follow-up period, data will also be collected on the number, if any, of individuals commenced on IPT who develop TB disease (Fig. 3).

Data on variables with the potential to affect outcomes will be collected and classified as study-related activities (categorized according to the COM-B model), external activities (e.g., introduction of a new recommendation by the provincial health authority), and environmental influences (e.g., holiday periods, major weather events, political unrest).

#### Study population

The study population for the quantitative data collection comprises TB index cases (people diagnosed with pulmonary TB) and their household contacts, using the WHO definition of a household contact [5, 41]. When the index case commences TB therapy, or later during treatment if the initial opportunity was missed, they will be asked to provide a list of household members and will receive an explanation that the public health team at the PHC will visit the household for contact investigation and management.

### Estimated sample sizes for contact investigation and preventive therapy

Numbers are provided in Table 3 showing the following minimum estimates: index case numbers per year, number likely to have contact investigation undertaken during the study period, number of contacts reached, number of contacts eligible for commencing and completing IPT, estimated number of cases of TB disease found during active case finding, and estimated number of TB cases averted. In Mimika, there are at least 1500 adult cases of TB notified annually, 1000 of whom are estimated to be managed at the five primary participating sites. We estimate up to five contacts per household, giving 10,000 individuals eligible for contact tracing over 2 years (Fig. 3).

We estimate that 40% of contacts (*n* = 4000) will be reached overall (aiming to progressively increase the two-year intervention from the baseline of zero contacts being reached); 5% of contacts reached will have co-prevalent active TB identified (*n* = 200); 15% of contacts reached will be eligible for TB preventive therapy (*n* = 600); 60% of those will commence on IPT (*n* = 360); 60% of those commencing IPT will successfully complete the 6-month regimen (*n* = 216) (Fig. 3). If the number needed to treat and to prevent a TB case is 35 [6], then for 216 people completing IPT, 6 TB cases will have been prevented.

These estimates are derived from previous studies of co-prevalent and incident tuberculosis and IPT [6, 11, 42, 43]. The proportion of child household contacts identified as having clinically or microbiologically diagnosed active TB during 12-month investigation periods was 7.8% in an Indonesian study [11] and 10% in an Ugandan study [42]. The proportion of all (adult and child) contacts with microbiologically confirmed TB in a household study in India was 6.9%, with about two thirds of these cases being co-prevalent—that is, picked up on initial household screening—and one third developing during the following 12 months (IPT was not given) [43]. This is why 5% has been estimated as the proportion of contacts who will be found to have co-prevalent active TB. The proportion eligible for IPT (no active TB and child aged < 5 or an immunosuppressed person of any age) is derived from the Indonesian study [11].

These numbers will provide good power to observe trends in clinic performance over time relating to child contacts being screened and treated appropriately and to undertake before-and-after calculations.

### Data analysis

**Fig. 4:**
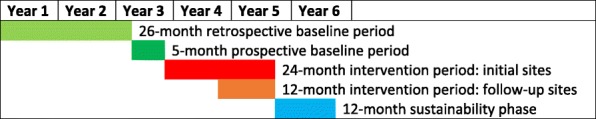
Timeline

Interrupted time series analysis will be used to analyze longitudinal time-dependent data. Study periods comprise a 26-month retrospective baseline period from 1 January 2015 to 31 March 2017, a 5-month prospective baseline period with active data cleaning and validation, a 24-month intervention period (including activities at initial and follow-up sites), and a 12-month sustainability period (Fig. 4). These periods were chosen pragmatically on the basis of available data and timing of study commencement. An annotated line graph, created with quarterly data points, will be produced to show the primary outcome (numbers of people completing IPT) over time, with the timing of key study activities, external activities, and environmental influences being indicated on the graph.

Segmented regression will be used to measure immediate changes in the rate of the outcome as well as changes in the trend. Calculation of rates will include 95% confidence intervals for the point estimates of rates in each time period. Crude rate will be calculated separately for intervention sites and non-intervention sites. It is assumed that populations of children in close contact with a TB patient in the intervention and non-intervention sites are comparable. The difference in rates will be calculated to estimate how the change in the intervention population differed from the change in the control population over the same time period.

AutoRegressive Integrated Moving Average (ARIMA) modeling will be used to test for any changes following the implementation of the program. Stationarity will be assessed and confirmed using the Augmented Dickey-Fuller (ADF) test on the log-transformed and re-plotted data. The data will be judged stationary with the *p* value of the ADF test < 5% level of significance. To choose the *p* and *q* parameters of the ARIMA, two diagnostic plots, i.e., autocorrelation function (ACF) and partial autocorrelation function (PACF), will be created as bar charts. The 95% confidence intervals will be shown as horizontal lines, and bars that cross these confidence intervals are therefore worth noting. The estimated value of *p* and *q* is the last lag with a large value if the autocorrelations (*q*) and partial autocorrelations (*q*) cut off after a few lags. A mixed model is suggested when neither the autocorrelations nor the partial autocorrelations cut off. Diagnostic checking of the model will be done once the model has been fitted.

### Qualitative study component

#### Study population

The study population for the qualitative component will be policymakers (employees of the District Health Office), healthcare providers, allied health practitioners, laboratory technicians, TB patients, parents of child contacts, and adult contacts of TB patients.

#### Methodological approaches

Qualitative data in the form of interviews, focus group discussions, and diarized observation will be collected by the doctoral researcher (TL), assisted by a trained research assistant.

*Semi-structured interviews* will be undertaken with TB patients, parents of child contacts, and adult contacts of TB patients. An interview guide will be developed on the basis of the theoretical frameworks informing this study—the CFIR and RE-AIM—to gain understandings of relevant barriers and facilitators of contact tracing implementation from the perspectives of providers and clients, and to gain information on their experience of participating in the project. TB patients and their contacts may wish to participate in group interviews rather than one-on-one interviews.

*Focus group discussions* with policymakers, healthcare providers, allied health practitioners, and laboratory technicians will be conducted as part of the continuous quality improvement cycles conducted every 3 to 4 months during the 24-month intervention period. Discussion topic guides will be developed, and written notes of these discussions will be taken and analyzed.

*Informal observations and conversations* on contact management will be made at the participating sites, and notes of these de-identified observations, including discussion in a social media platform described below (“WhatsApp™”), will be used for analyses.

Individual written, informed consent to undertake and record interviews and group discussions will be sought. This will be provided in Bahasa Indonesia, or verbally in a relevant Papuan language. If the study participant cannot sign, a thumbprint will be used to signify consent. Parental consent will be sought for interviews of any TB contacts or index cases aged < 18 years, with additional assent from the subject themselves. Purposive sampling with a maximum variation approach will be employed to select participants [44]. Targeted numbers are shown in Table 4. This sample size provides a feasible number likely to provide an adequate range of opinions, expertise, and themes. However, considering issues of qualitative research emergent design and data saturation, these estimated sample sizes may be subject to change.

**Table 4:** Qualitative data collection: sample sizes and target populations

Planned sample sizes for qualitative data collection	
Health care providers	15
Allied health staff	3
Policy makers	7
TB patients	25
TB contacts or their parents	25
Considerations in selection of study participants	
Healthcare providers	
Health institution where participant is employed	Primary health care facility, hospital, district health office, local TB NGO, other TB care provider (clinic, private pharmacy)
Experience with TB	< 5 years, ≥ 5 years
Contact with TB patient	Direct contact (e.g., clinicians) or indirect contact (e.g., laboratory workers)
Knowledge and Training	Trained or not trained in child TB management
Sex	Male and female
Ethnicity	Papuan and non-Papuan
Patients and families	
Education	High school, primary school, or minimal/none. Education level in the family measured by asking level of education of each family member and selecting the highest
Occupation	Formal and informal sector measured by asking occupation of the family member in productive age
Number of families living in the same house	Single family Multiple families
Place of living	Timika City, low-land areas, highland areas
Sex	Male and Female
Ethnicity	Papuan and non-Papuan

Qualitative data will be transcribed by study nurses, translated into English prior to analysis, and organized in NVivo qualitative data analysis software (QSR International Pty. Ltd., Version 10, 2014). Interview data will be analyzed using qualitative content analysis. This method allows a focus on selected aspects of meaning instead of opening the data to wide interpretation. This is done systematically by assigning relevant parts of interview contents to a pre-specified coding frame, built from the theoretical frameworks (CFIR, COM-B, and RE-AIM) and grouped into categories and sub-categories. The data collection (interviews, focus group discussions, observations, and interviews) and coding and categorizing process will be continued until saturation is reached, that is, until no additional new meaning can be found. Coding consistency will be assessed by comparing two rounds of coding carried out by independent coders (TL and CvdB). When all categories have been generated, cleaned, and revised, the codes will be structured and narrated to answer the research questions. Text matrices will be made to identify differences between sources or co-occurrences between categories [45].

### Process evaluation: Theories and frameworks

Formative evaluation (evaluation done during project development and implementation to improve ongoing activities) is being undertaken using the CFIR [32]. This process commenced during baseline scoping activities to understand the local context including barriers to implementation of the contact management policy and will continue as qualitative data are collected. The CFIR framework is a synthesis of 19 existing implementation theories [31]. It describes five domains which we have interpreted as relevant to this project as follows: (1) *intervention*—activities to support behavior change among policymakers, healthcare providers, and patients to adopt the TB contact management policy; (2) *outer setting*—the financial and sociopolitical environment of Mimika health services; (3) *inner setting*—management issues at participating hospitals and PHC and factors relating to the research funding and governance; (4) *individuals*—people with key roles in the project at the District Health Office, participating PHCs and hospitals, and patients and their families); (5) *implementation process—*how the activities are rolled out (fidelity to the study protocol) and the development of collaborative relationships.

Alongside the CFIR, the theory used to develop the intervention components is the COM-B model [24]. This model posits that the requirements to change behavior are capability, opportunity, and motivation. To provide capability, opportunity, or motivation to change behavior, interventions (such as education and/or incentives) are needed, which must operate within context-specific policy considerations (e.g., legislative factors). Together these three layers—“COM-B,” “intervention functions,” and “policy considerations”—form the Behaviour Change Wheel [24]. Preliminary information obtained during project planning was categorized according to the CFIR domains and mapped against the Behaviour Change Wheel (Table 5). This provides an initial understanding of the main barriers which need to be addressed for successful policy rollout.

**Table 5:** Selected examples of barriers and facilitators identified using the CFIR mapped to the BCW

Consolidated Framework for implementation Research (CFIR)		Behaviour Change Wheel (BCW) components									
Domain of CFIR (domains 1 to 4)	Barriers and facilitators identified using CFIR		Education	Persuasion	Incentives	Coercion	Training	Restriction	Environmental structuring	Modeling	Enablement
(1) Individuals	Knowledge about TB contact investigation and preventive therapy	Influencing capability	X	X			X				
(3) Inner setting	Perceived risk of TB transmission during house visit	Influencing capability and motivation	X	X			X		X		
(2) Outer setting	Language barriers	Influencing opportunity							X		X
(3) Inner setting	Availability of isoniazid for children	Influencing opportunity		X					X		X
(2) Outer setting	Distance to patient’s house	Influencing motivation	X	X	X				X		X
(1) Intervention	Duration of treatment	Influencing motivation	X	X	X						
(4) Implementation process	Delay in identifying child contacts	Influencing capability and motivation	X				X		X	X	X

The evaluation framework we selected is RE-AIM (Reach, Effectiveness, Adoption, Implementation, Maintenance) [32]. This provides a way to describe how the project will be implemented and evaluated and why it will be expected to be successful. The RE-AIM model provides a framework to comprehensively measure public health impact of research conducted in real-world settings rather than controlled trial settings [46, 47]. This is highly suited to our pragmatic intervention. The RE-AIM model consists of five dimensions, occurring in a logical sequence: adoption, reach, implementation, effectiveness, maintenance. Only a small proportion of studies address each dimension and use qualitative inquiry to interpret findings, which can lead to more effective research translation [46]. We address each dimension of the RE-AIM model as described in Table 6.

**Table 6:** Application of the RE-AIM framework to this project

RE-AIM dimension and operational definition	Plan	Indicators
How will I and the intervention reach the targeted population? Reach is the absolute number or proportion of individuals who are willing to participate in a given initiative.	• Project staff will visit clinics directly and meet with relevant local stakeholders. We already have support and permission from relevant local authorities to undertake the study. • TB staff at the participating health facilities will be trained in workshops and informal on-the-job interactions to undertake the intervention, comprising identifying TB symptoms and asymptomatic TB contacts, referral, education of patients, prescribing, and monitoring. • Project staff will also have direct contact with selected, consenting TB patients and their contacts through referral by clinic staff, for interviews and provision of education.	• Proportion of health providers who participate in the intervention • Uptake of contact investigation among smear sputum positive TB individual • Uptake of preventive therapy among child contact of smear sputum positive TB cases
How do I know my intervention is effective? Effectiveness is the impact of an intervention on outcomes, including potential negative effects, quality of life, and economic outcomes.	• Relevant data will be collected regularly. The listed quantitative outcome measures will demonstrate whether interventions are working • Results of staff interviews will show whether staff have adequate knowledge of the process of contact management, and whether they are satisfied with the project.	• Significant change in the practice of contact investigation and preventive treatment identified using ARIMA model • Number of new TB cases (adult and children) identified from contact investigation • Number of children eligible for preventive therapy identified from contact investigation • Number of eligible children commence on preventive therapy • Adherence to preventive therapy among eligible contacts
How do I develop organizational support to deliver my intervention? Adoption is the absolute number, proportion, and representativeness of settings and intervention agents who are willing to initiate a program.	• This has been achieved already through face-to-face meetings with and endorsement from the Timika Health Authority (Dinas Kesehatan Timika), as well as endorsement of the study protocol by the National TB expert committee. A partnership agreement has been signed with all selected health facilities. • Number of healthcare workers who were trained and actively participated in the program will be measured.	• Health staff perception about the appropriateness, comfort, relative advantage, and credibility of the intervention • Proportion of TB nurses, TB physicians, head of Puskesmas, hospital managements, and district health office staff who are willing to accept, support. and participate in the intervention • The extent of support and participation of TB-related health staff and stakeholders in the contact investigation and preventive therapy program
How do I ensure the intervention is delivered properly? Implementation refers to the intervention agents’ fidelity to the various elements of the intervention’s protocol	• Project staff will directly supervise project implementation. The research team (study nurses working with doctoral researcher) has been well trained and already has a strong background in TB management. • Data checking will be undertaken regularly during the project.	• Fidelity of the intervention • The extent to which the intervention can be carried out in Puskesmas and Hospital • Gap between indigenous and non-indigenous Papuan people in the access and quality of the intervention
How do I incorporate the intervention, so it is delivered over the long-term? Maintenance is the extent to which a program or policy becomes institutionalized or part of the routine organizational practices and policies.	• The use of continuous quality improvement will embed practice locally and sustainably. • Some actions to repair logistic barriers to TB contact tracing (e.g., lack of medication supply) are being overcome early in the study—this effect will be sustained. • Train-the-trainer concepts will be employed to ensure sustainability of knowledge within healthcare centers. • Inclusion of budget for isoniazid prevention therapy and contact investigation in primary care facilities or hospital budgets can also be indicators of program sustainability.	• The extent to which the intervention is institutionalized or maintained within health facility and district health system • Integration of the intervention to the other existing program in health facilities.

*Puskesmas* pusat kesehatan masyarakat (community health center)

### Intervention components

During the 24-month intervention phase, a suite of interventions will be implemented in a staggered way at the five initial participating sites. Activities will be led by the doctoral research student, supported by the study nurses, a “regional research champion” (Indonesian TB expert), and study investigators. Once policy implementation is well embedded, selected healthcare providers at these sites will be invited to participate in train-the-trainer sessions, so they can disseminate information about the policy at the second-tier (follow-up) sites. The two-tiered approach to the intervention will provide a further measure of sustainability, in addition to the 12-month sustainability period. Intensive activities will take place with the five initial participating sites, then healthcare providers at these sites will then be trained (using a “train-the-trainer” approach) to share their knowledge with the seven follow-up sites.

#### Training

Training will be provided by the doctoral research student and Indonesian TB experts, in the form of workshops for healthcare providers from the five primary participating sites, and any additional healthcare providers from other sites who wish to attend (including the follow-up sites). Training sessions will be free of charge and during usual working hours, by arrangement with management at their respective workplaces. The elements of TB contact management policy will be taught, including practical implications and logistics of rolling this out at each clinic. Hands-on training will be provided in the skills required for policy implementation, such as collection of induced sputum samples from those found to have TB symptoms and provision of accurate weight-based dosing of isoniazid. Pre- and post-training tests will be conducted to understand knowledge and measure the effectiveness of training.

#### Continuous quality improvement

Continuous quality improvement (CQI) forms a cornerstone of implementation of this project at the five initial sites. CQI cycles will be implemented whereby a quarterly workshop will be held to allow staff at the five sites to engage proactively with their data. Plots and pictograms will be generated that are easy for healthcare providers to understand, showing progress in key performance indicators, such as the proportion of contacts investigated and the proportion of eligible contacts commenced on IPT. Discussions to congratulate and motivate staff will be undertaken, and challenges will be workshopped. Where appropriate, recommendations arising from these CQI workshops to address challenges in policy implementation (e.g., staffing allocations, access to transport for outreach services) will be communicated to the District Health Authority. The approach will draw on lessons learned in previous research using CQI cycles in primary care to improve guideline adherence [48–50].

#### Mentoring and clinical support

Mentoring and clinical support will be provided directly, through face-to-face discussions during site visits and telephone calls via a WhatsApp™ group. The research team will visit study sites at least weekly to ask about program updates, current activities, and challenges and respond to questions related to program implementation. The research team can also be telephoned at any time as needed. Healthcare providers participating in the study at both initial and follow-up sites will be invited to a WhatsApp™ group moderated by the doctoral research student and with access to an Indonesian TB specialist. WhatsApp™ Messenger is the most widely used, freely available communication application in Indonesia, accessible from mobile devices or desktop computers. This will provide a forum for information sharing relevant to the study, and participants will be able to post questions relating to the contact management policy or other aspects of TB care.

#### Awareness-raising activities and promotional materials

Posters and information brochures about contact investigation and preventive treatment in two languages—one of the main local Papuan languages and Bahasa Indonesia—have been created for the study and made available at participating facilities. A picture of lungs showing the differences between latent TB infection, early pulmonary TB, and severe pulmonary TB will be used by TB staff to help explain the importance of TB prevention.

## Discussion

This study proposal addresses key pillars of the End TB strategy, namely “integrated patient-centered care and prevention” (pillar 1) and “research to optimize implementation and impact” (pillar 3) [3], which are currently underimplemented across high-burden settings. While many aspects of this study plan are specifically tailored to the Mimika context, most components are highly relevant to Indonesia as a whole and high-burden, low-resource settings more broadly.

We considered a range of study designs for this intervention. A community cluster randomized trial (either single-phase or stepped-wedge design) could have been employed, but these designs have limitations. Both require significant resources; a cluster randomized trial in this context could be particularly subject to contamination due to the extent of communication between healthcare providers at healthcare facilities and between the district health authority and healthcare facilities, and there would be ethical implications if the international standard of care (IPT) was not implemented at control sites. A stepped-wedge design might not provide adequate time for interventions to have a maximal effect [48]. We chose a design that was pragmatic and replicable. To test sustainability, a maintenance phase has been included, and the two-staged rollout design will provide an indication of whether local staff using existing resources will be effective in promoting policy implementation at follow-up sites.

The rationale for the use of a combination of theoretical frameworks (the CFIR, Behaviour Change Wheel, and RE-AIM) is that the CFIR is an overarching approach explicitly recommended for use in combination with process and impact theories [31]. It provides a systematic approach for articulating barriers to project implementation. The Behaviour Change Wheel is a well-validated way to then ensure that comprehensive responses to these barriers are provided during project implementation. Finally, an evaluation framework is required to report on process and impact, which is provided by the RE-AIM model, in which the investigator team has experience [51]. However, a potential limitation is that the application of such frameworks is open to the interpretation of study investigators, such that findings from different studies using the same frameworks may be difficult to synthesize [52].

The application of continuous quality improvement (CQI) cycles using routinely collected data is a way to engage clinic staff in understanding their own data to seek to motivate behavior change. We [50, 53] and others [54] have shown the effectiveness of this type of approach in achieving health system improvement. The approach is very applicable in this project where TB data are routinely collected and reported, but often not reflected upon, especially not in real time. Increasing effectiveness of CQI cycles appear to be associated with longer duration of participation by healthcare facilities in this process [49]; therefore, in 2 years of implementation, we should see the impact but may not be able to realize maximal scale up during that time.

Initial implementation of this project is underway. Challenges in this low-resource setting are formidable. Some challenges such as the distance between a patient’s house and the clinic, and others summarized in Table 5, are universal [7, 13, 14, 16]. Others are context specific. Within the first months of the study, two healthcare facilities in the district were the subject of arson attacks. Internet failures are commonplace, and petrol shortages occur. Procurement of essential supplies for the TB program is patchy, with frequent stock-outs of basic supplies. However, a key facilitating factor is that the District Health Authority is highly supportive of this project. Factors which particularly appeal to the authority include that the project directly seeks to improve locally important reporting targets; success in the study will reflect well for the district and allow it to be considered a model for other districts to follow. Also, data in the study will be directly visible as they arise, in contrast with other designs such as blinded trials in which data are unobtainable until study completion. This will allow the District Health Authority to feel engaged and importantly provide the necessary channel for knowledge translation including project upscaling.

A specific challenge at study commencement was that low-dose isoniazid suitable for pediatric dosing was unavailable throughout the province. The minimum tablet dose was 300 mg, whereas the dose for infants is 10 mg/kg per day—hence ranging from 50 to 100 mg for an infant. Tablets would need to be divided into small fractions to achieve the correct dose. An early success as a result of relationship building with District and Provincial Health Authorities in Mimika and National TB program was the procurement and distribution of 100 mg isoniazid tablets throughout the district.

Initial data indicate that the majority of TB cases are diagnosed at hospitals rather than PHCs, but it has quickly become apparent that hospitals have very limited capacity to undertake contact management since they lack public health staff to conduct home visits. Contact management can occur in healthcare facilities, but only if families can afford time away from work and transport costs required to attend; these costs are too great for the majority of TB patients in Mimika. Therefore, in this setting, as in comparable international settings [16], contact management is best done in households, but this requires adequate resourcing. Creative solutions are needed and are being considered in the delivery of this project: ensuring that hospitals refer patients back to PHCs for TB care, to facilitate contact management; considering assistance with transport costs provided in a sustainable way through the TB Program; or partnering with non-government organizations (NGOs) who could assist in providing contact management for hospital patients.

Several potential limitations of the study are foreseeable. A possible challenge in the analysis of findings will arise in deciding how any changes seen can be attributed to study activities compared with external coincident factors. This will be managed by ensuring that the timing and nature of activities are well documented. Qualitative data obtained from interviews will also provide insights into how or why healthcare providers and clients might change their behavior and practice. A second potential limitation is the sustainability and the ability to measure this. While the study requires a large amount of input from the doctoral researcher, the study nurses, and the investigator team, the expectation is that after setting the policy in motion, implementation would become sustainable. The two-tier study design, resembling a ripple effect, puts in place a system to support sustainability by training local people to becomes advocates for the policy, and trainers of others. A third major factor which could challenge the overall success of the project is the local socioeconomic and political situation. Poverty and limited resources at all levels of healthcare systems limit the ability of healthcare providers to provide best-practice care and for clients to participate in care. Creative strategies to work efficiently within existing resources and a cost-effectiveness mentality (upfront investment, e.g., in outreach to find TB contacts, to prevent new active TB cases) will be encouraged. Ensuring that the project is locally led and maintaining good communication with local, regional, and national stakeholders will optimize the chance of success despite this sometimes-volatile environment.

## Conclusion

This study protocol describes a pragmatic implementation research approach to the rollout of TB contact investigation and management. It is tailored to the local setting but with wide application nationally and internationally in resource-constrained high TB-burden settings.

## Abbreviations

CFIR: Consolidated Framework for Implementation Research
COM-B: Capability, Opportunity, and Motivation Behavior
CQI: Continuous quality improvement
HIV: Human immunodeficiency virus
IPT: Isoniazid preventive therapy
MDR: Multidrug resistant
NTP: National TB Program
PHC: Primary health center
RE-AIM: Reach, Effectiveness, Adoption, Implementation, Maintenance
TB: Tuberculosis
WHO: World Health Organization
